# The role of the JAK2-STAT3 pathway in pro-inflammatory responses of EMF-stimulated N9 microglial cells

**DOI:** 10.1186/1742-2094-7-54

**Published:** 2010-09-09

**Authors:** Xuesen Yang, Genlin He, Yutong Hao, Chunhai Chen, Maoquan Li, Yuan Wang, Guangbin Zhang, Zhengping Yu

**Affiliations:** 1 Key Laboratory of Medical Protection for Electromagnetic Radiation Ministry of Education, Third Military Medical University, Chongqing 400038, China; 2 Institute of Tropical Medicine, Third Military Medical University, Chongqing 400038, China; 3 International Travel Healthcare Center, Chongqing Entry-Exit Inspection and Quarantine Bureau, Chongqing 401147, China

## Abstract

**Background:**

In several neuropathological conditions, microglia can become overactivated and cause neurotoxicity by initiating neuronal damage in response to pro-inflammatory stimuli. Our previous studies have shown that exposure to electromagnetic fields (EMF) activates cultured microglia to produce tumor necrosis factor (TNF)-α and nitric oxide (NO) through signal transduction involving the activator of transcription STAT3. Here, we investigated the role of STAT3 signaling in EMF-induced microglial activation and pro-inﬂammatory responses in more detail than the previous study.

**Methods:**

N9 microglial cells were treated with EMF exposure or a sham treatment, with or without pretreatment with an inhibitor (Pyridone 6, P6) of the Janus family of tyrosine kinases (JAK). The activation state of microglia was assessed via immunoreaction using the microglial marker CD11b. Levels of inducible nitric oxide synthase (iNOS), TNF-α and NO were measured using real-time reverse transcription-polymerase chain reaction (RT-PCR), enzyme-linked immunosorbent assay (ELISA) and the nitrate reductase method. Activation of JAKs and STAT3 proteins was evaluated by western blotting for specific tyrosine phosphorylation. The ability of STAT3 to bind to DNA was detected with an electrophoresis mobility shift assay (EMSA).

**Results:**

EMF was found to significantly induce phosphorylation of JAK2 and STAT3, and DNA-binding ability of STAT3 in N9 microglia. In addition, EMF dramatically increased the expression of CD11b, TNF-α and iNOS, and the production of NO. P6 strongly suppressed the phosphorylation of JAK2 and STAT3 and diminished STAT3 activity in EMF-stimulated microglia. Interestingly, expression of CD11b as well as gene expression and production of TNF-α and iNOS were suppressed by P6 at 12 h, but not at 3 h, after EMF exposure.

**Conclusions:**

EMF exposure directly triggers initial activation of microglia and produces a significant pro-inflammatory response. Our findings confirm that the JAK2-STAT3 pathway may not mediate this initial microglial activation but does promote pro-inflammatory responses in EMF-stimulated microglial cells. Thus, the JAK2-STAT3 pathway might be a therapeutic target for reducing pro-inflammatory responses in EMF-activated microglia.

## Background

The rapid development of society and technology has led to an unprecedented increase in the number and diversity of sources of electromagnetic fields (EMFs), including power lines, electric appliances, radio transmitters, and microwave sources. Numerous studies have investigated the effects of occupational or residential exposure to EMF, which has been identified as the fourth largest pollution hazard [1-7]. Several studies have suggested that biological systems exhibit a specific sensitivity to 2.45 GHz microwaves (a water resonance frequency), which is widely used in household appliances, medical applications and communication systems. While some of these results have been difficult to reproduce, or have severe methodological shortcomings [8,9], other studies have reported that occupational exposure to EMF may be associated with gliomas [10-12] or Alzheimer's disease [13-15]. Therefore, the effects of EMF exposure on the central nervous system (CNS) have been an active topic of investigation in recent years. Several studies have revealed strong glial reactivity in different parts of the brain after EMF exposure [16-19]. We have found activated microglia in the hippocampus and cortex of rats after exposure to EMF (unpublished results). *In vivo *animal experiments involving microglial activation, however, cannot clearly explain whether such activation is induced directly by EMF or indirectly as a consequence of neuronal injury from EMF exposure.

Microglia, the resident innate immune cells in the CNS, become activated in response to certain cues, such as brain injury and immunological stimuli [20,21]. Activated microglia undergo a dramatic morphological transformation. They then become motile and acquire a reactive profile that is characterized by proliferation, migration and phagocytosis [22-28]. Overactivated microglia can result in disastrous and progressive neurotoxic consequences, however, leading to excess production of factors such as superoxide [29], nitric oxide (NO) [30-32] and tumor necrosis factor-α (TNF-α) [33-36] that cause additional neuroinflammation [37-40]. Not surprisingly, activated microglia are important in the pathogenesis of neurodegenerative diseases, such as Alzheimer's disease [41], Parkinson's disease [42,43] and amyotrophic lateral sclerosis [44-46].

The signal transduction mechanisms involved in microglial activation and neuroinflammatory factors release after EMF exposure are still largely unknown. Microglia may be the principal target of the neurobiological effects of EMF. In response to extracellular stimuli, several major signaling pathways are upregulated in activated microglia. Several transcription factors, *i.e.*, NF-κB, AP-1 and C/EBP, are involved in microglial activation *in vivo *and *in vitro *[47-49]. STAT signaling is another critical pathway that plays an important regulatory role in microglial reactivity to various stimuli, including cerebral ischemia, gangliosides, lipopolysaccharide, thrombin and cytokines [50-55]. We have previously shown that the JAK-STAT3 pathway is activated in EMF-stimulated microglia [56]. It is not known, however, whether JAK-STAT3 signaling triggers the initial activation of EMF-stimulated microglia or whether it merely participates in the pro-inflammatory responses. Recently, a JAK inhibitor I (pyridone 6, P6), capable of producing complete inhibition of STAT3 activation, was shown to not alter the growth characteristics of tested cell lines even when used in a high μM range of concentrations. This observation suggests that persistent STAT3 inhibition with P6 may be a helpful tool in addressing the aforementioned questions.

Imbalanced microglial activation or hyperactivation can cause neurodegeneration, but the true initial trigger(s) of microglial activation has not been identified [57]. Furthermore, the role of the JAK-STAT signaling pathways in EMF-stimulated microglia has not yet been investigated. Hence, we exposed cultured N9 microglia to 2.45 GHz electromagnetic fields and examined microglial activation, the release of pro-inflammatory factors, and the role of the JAK-STAT signaling pathway in this process.

## Methods

### Cell culture

The mouse microglial cell line N9 was a gift from Dr. Bai Yun (Department of Genetics, The Third Military Medical University, China) and was cultured as described in the original publications [58,59]. Brieﬂy, cells were grown in Iscove's modified Dulbecco's medium (IMDM; HyClone, Logan, UT) supplemented with 5% heat-inactivated fetal bovine serum (FBS; HyClone), 2 mM glutamine, 100 U/ml penicillin, 100 μg/ml streptomycin, and 50 μM 2-mercaptoethanol (Sigma-Aldrich, St. Louis, MO). Cells were seeded in 25-cm^2 ^T-flasks (1×10^6 ^cells/flask) or 6-well plates (5×10^5 ^cells/well) at 37°C in a humidified 5% CO_2 _atmosphere. The medium was exchanged for serum-free IMDM after 24 h. Cells were then pretreated with or without P6 (10 μM; Calbiochem, La Jolla, CA) or a solvent control (tissue culture grade dimethylsulfoxide (DMSO; Sigma-Aldrich)) for 1 h prior to EMF stimulation.

### Exposure system

Pulsed EMF exposure was carried out in an anechoic chamber, and the ambient air temperature inside the anechoic chamber was 25-26°C. Pulsed EMF was delivered through a rectangular horn antenna connected horizontally to a handset (Philips PM 7320X). The radiation was directed vertically downward toward the exposure flasks using a reflector. The microwave transmitter was operated at 2.45 GHz at an average pulsed power of 90 mW. The pulse width was 2 μs, and the pulse repetition rate was 500 pps. A 20-min exposure to 2.45 GHz pulsed microwaves at an average specific absorption rate (SAR) of 6 W/kg was performed. During the 20-min exposure period, the distance from the face of the antenna horn to the surface of the flasks where the cells were settled was 90 cm. For EMF exposure, four flasks were placed into the upper chamber of a Perspex™ water bath (24.5 × 21 cm). The temperature of the medium in the flasks in the upper chamber was maintained at 37°C by circulating heated water through the lower closed chamber. During sham exposure, four T-25 flasks were placed in the same conditions for the same period of time as the EMF-exposed group, except for the EMF exposure. Finite difference time domain (FDTD) analysis was performed to calculate the SAR value [60].

### Enzyme-linked immunosorbent assay (ELISA) of TNF-α

TNF-α release in cultured supernatants was determined using a mouse TNF-α ELISA kit (eBioscience, San Diego, CA). Briefly, ELISA plates (96-well; NUNC MaxiSorp, eBioscience) were coated with coating buffer (100 μl/well), sealed and incubated overnight at 4°C. The wells were washed 5 times with wash buffer and blocked with assay diluent at room temperature for 1 h. The samples (100 μl) collected from N9 cultures were added to each well and incubated overnight at 4°C for maximal sensitivity. Subsequently each plate was incubated with the detection antibody (100 μl/well) diluted in assay buffer for 1 h and then avidin-HRP (100 μl/well) diluted in assay diluent for 30 min at room temperature. Each plate was subsequently incubated with tetramethylbenzidine (TMB) substrate solution for 15 min; the reaction was stopped with 50 μl of 2 N H_2_SO_4 _stop solution. The results were read using a microplate spectrophotometer at 540 nm. A standard curve prepared from recombinant TNF-α was used to calculate the TNF-α production of the samples.

### Nitric oxide determination in culture medium

NO was measured as released NO metabolites (nitrates and nitrites) using an NO detection kit (Nanjing Jiancheng Bioengineering Institute, Nanjing, CN). This method uses nitrate reductase to specifically reduce NO^3- ^to NO^2-^, and the content of NO^2- ^is determined colorimetrically. Briefly, 100 μl of incubation medium and a standard were added to the wells. Then, 50 μl of nicotinamide adenine dinucleotide (NADH) and nitrate reductase was added. After 30 min, 100 μl of Greiss reagents I and II was added and incubated for 10 min at room temperature. The optical density of each well was determined using a microplate reader set at 540 nm.

### Flow cytometry

Expression of microglial marker CD11b [61] was measured by fluorescence-activated cell sorting (FACS) analysis to assess activation state of microglial cells. Briefly, after 20 min of EMF or sham exposure, microglial cells were washed three times with flow buffer (phosphate-buffered saline (PBS) containing 0.1% (w/v) sodium azide and 1% (w/v) BSA) and re-suspended in 250 μl of ice-cold flow buffer. Cells were pre-incubated with goat serum (Zhongshan Goldenbridge Biotechnology (Zsbio), Beijing, CN) for 20 min at 4°C to block non-specific binding to Fc receptors. Cells were then spun down at 5,000 rpm, washed three times with flow buffer, and incubated with rat anti-mouse monoclonal antibody CD11b (1:100; AbD Serotec, Oxford, UK) or rat IgG2b isotype control (1:100; AbD Serotec) for 1 h at 4°C. Centrifugation and washing steps were repeated, and cells were then incubated with goat anti-rat IgG-DyLight^®^549 (1:200; AbD Serotec) for 1 h at 4°C in the dark. Quantitative analysis was performed using a FACSCalibur system (BD Biosciences, San Jose, CA).

### Confocal microscopy with double-label immunofluorescence

As previously described [62], cultured cells were fixed and permeabilized. Cells were then pre-incubated with goat serum (Zsbio) for 20 min at room temperature and then washed 3 times with flow buffer. For immunoﬂuorescence labeling, cell cultures were incubated with one of the following antibodies for 1 h at 37°C: rat anti-mouse monoclonal antibody CD11b (1:100; AbD Serotec) and rabbit anti-mouse monoclonal pTyr705-STAT3 antibodies (p-STAT3, 1:100; Cell Signaling Technology, Danvers, USA). For confocal microscopy of the double-labeled samples, cell cultures were incubated simultaneously with goat anti-rat IgG-DyLight^®^549 (1:200; AbD Serotec) and sheep anti-rabbit IgG-FITC (1:200; Sigma-Aldrich, St. Louis, USA) for 1 h at 37°C in the dark. Cell cultures were then washed and mounted with aqueous-based anti-fade mounting medium. Images of stained cells were captured using a Leica TCS-SP5 confocal laser scanning microscope (Leica, Mannheim, Germany). Image analysis was performed with a semi-quantitative method. Fluorescence intensity was measured using software Image J 1.42.

### Western blotting

Cells were washed with ice-cold PBS and scraped in RIPA lysis buffer (Roche, Penzberg, Germany) containing protease inhibitors. Whole cell extracts (80 μg/lane) were separated by 8% SDS PAGE under reducing conditions and then transferred onto nitrocellulose membranes (Millipore, Bedford, USA). The membranes were blocked with a special Odyssey blocking buffer (LI-COR, Lincoln, USA) for 3 h at room temperature. The proteins were detected by incubation at ambient temperature with monoclonal antibodies for p-STAT3 (1:1000; Cell Signaling Technology), STAT3 (1:1000; Cell Signaling Technology), phospho-JAK1 Tyr-1022/1023 (p-JAK1, 1:2000; Santa-Cruz Biotechnology, Santa Cruz, CA), JAK 1 (1:1000; Cell Signaling Technology), phospho-JAK2 Tyr-1007/1008 (p-JAK2, 1:2000; Santa-Cruz Biotechnology), and JAK2 (1:1000; Cell Signaling Technology) for 3 h. Membranes were washed four times for 5 min each in tris-buffered saline Tween-20 (TBST) and then incubated with a fluorescently-labeled secondary antibody (1:5000, LI-COR) for 30 min at room temperature with gentle shaking. After the final washes with PBS, the signal was detected and quantified with the Odyssey infrared imaging system (LI-COR). Loading controls were detected with mouse monoclonal anti-β-actin antibody (1:5000, Sigma-Aldrich).

### Electrophoresis mobility shift assay (EMSA)

The electrophoresis mobility shift assay (EMSA) for nucleoprotein extracts (10 μg) was performed using the Odyssey Infrared EMSA Kit (LI-COR) according to the manufacturer's instructions. The following double-strand oligonucleotides were used as specific labeled probes or cold competitors: 5'-GAT CCT TCT GGG AAT TCC TAG ATC-3', 3'-CTA GGA AGA CCC TTA AGG ATC TAG-5'. Nuclear extract and STAT3 IRDye™ 700 infrared dye-labeled oligonucleotides (LI-COR) were incubated according to the manufacturer's instructions. The mixture was incubated for 30 min at 30°C. Electrophoresis was performed at 10 V/cm at 4°C using 5% native polyacrylamide gels. The gels were scanned with the Odyssey scan bed (LI-COR).

### Reverse transcription-polymerase chain reaction (RT-PCR)

Total RNA was isolated using Trizol (1 ml/well; Roche, Penzberg, DE) from 6-well plates according to the manufacturer's protocol. The integrity of the RNA sample was confirmed with gel electrophoresis and by reverse transcription-PCR using primers for house-keeping genes. Moloney murine leukemia virus (M-MLV) reverse transcriptase (Toyobo, Tokyo, Japan) was used to convert 1 μg of total RNA into cDNA at 42°C. The RT-PCR exponential phase was determined to be 20-32 cycles for semiquantitative comparisons. β-Actin was measured as a loading control. The amplification of cDNA was performed with the following primers: ***mouse β-actin ***[GenBank: 007393.2]: forward 5'-TAAAGACCTCTATGCCAACACAGT-3'; reverse 5'-CACGATGGAGGGGCCGGACTCATC-3'. ***iNOS ***[GenBank: 010927.2]: forward 5'-AACTGTAGCACAGCACAGGAAA-3'; reverse 5'-ACAAGATCAGGAGGGATTTCAA-3'. ***TNF-α ***[GenBank: 013693.2]: forward 5'-GCCTATGTCTCAGCCTCTTCTC-3'; reverse 5'-GGAGGTTGACTTTCTCCTGGTA-3'. The amplification reaction was carried out in a Perkin-Elmer GeneAmp (Perkin Elmer, Boston, USA). The resulting PCR products were separated in 1.5% agarose gels and visualized with ethidium bromide staining. For semi-quantitative evaluation, densitometric analysis was performed using Quantity One software (Bio-Rad, Hercules, USA).

### Statistical analysis

Data are presented as the mean of each treatment group ± standard deviation of the mean (SD). Statistical differences between the groups were assessed by one-way analysis of variance (ANOVA) followed by Duncan's Multiple Range test. Statistical significance was established at P < 0.05 unless otherwise indicated.

## Results

### Effect of EMF exposure on CD11b expression in N9 cells

**Figure 1:**
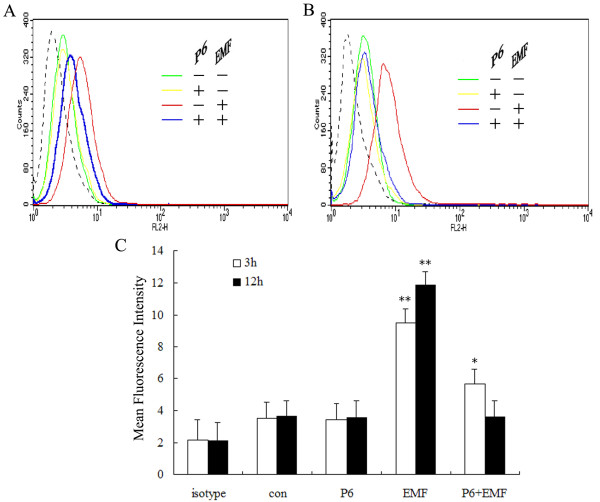
**Flow cytometry analysis of the effect of EMF exposure on CD11b expression in N9 cells**. Microglia were pretreated with (+) or without (-) P6 (10 μM) for 1 h and then exposed to 2.45 GHz EMF (+) or sham-exposed (-) for 20 min. Untreated cultures were used as a control. Cells were incubated with rat anti-mouse monoclonal antibody CD11b or isotype control for 1 h at 4°C and then incubated with goat anti-rat IgG-DyLight^®^549 for 1 h at 4°C in the dark. In total, 10,000 cells were labeled (CD11b; rat monoclonal, 1:100; rat IgG2b isotype control, 1:100; DyLight^®^549-conjugated goat anti-rat secondary, 1:200), gated and analyzed by flow cytometry. Histogram overlays show the expression of CD11b. The dashed lines represent the isotype control. (**A**) Representative histograms for CD11b expression at 3 h after EMF exposure. (**B**) Representative histograms for CD11b expression at 12 h after EMF exposure. (**C**) Mean fluorescence intensity of CD11b shows averaged values from three independent experiments as normalized to the control. CD11b expression was found to be significantly increased at 3 h after EMF exposure; a slight but significant increase was still apparent after P6 pretreatment. CD11b expression was found to be strongly expressed at 12 h after EMF exposure; this increase was significantly inhibited by P6 at 12 h after EMF exposure. Results are presented as mean ± S.D. of three independent experiments. Statistical comparisons to control are indicated by * p < 0.05; ** p < 0.01.

**Figure 2:**
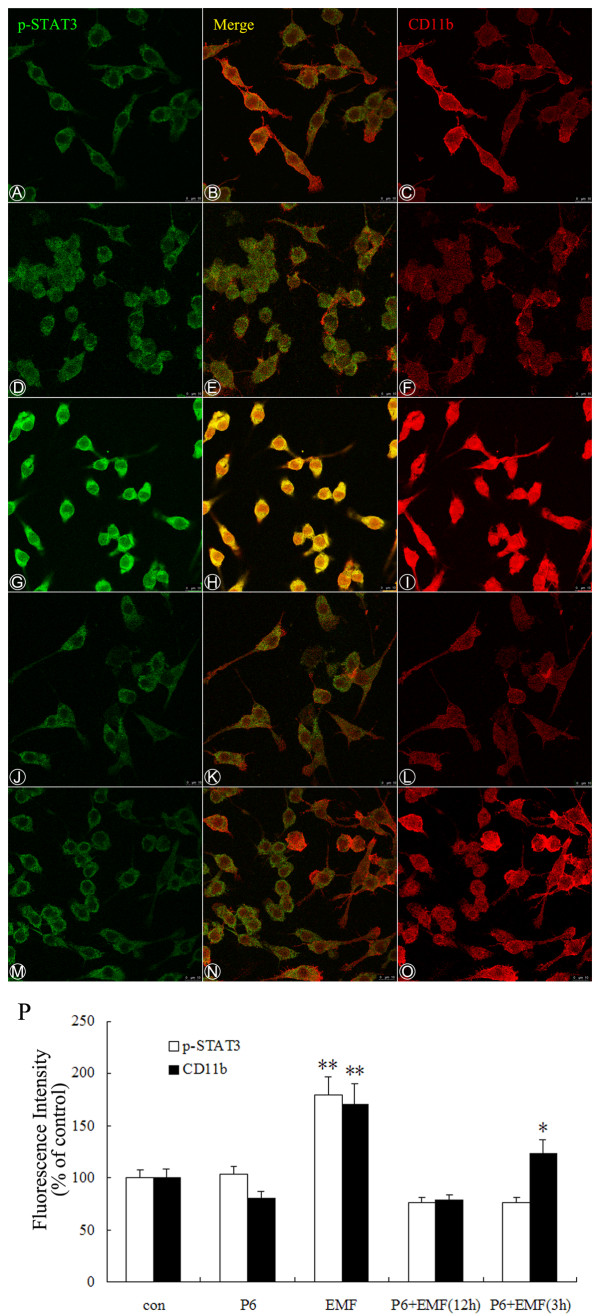
**Localization of CD11b and p-STAT3 immunoreactivity in activated N9 cells**. Experiments were performed as described above. Confocal immunofluorescence microscopy was performed on cultures that were immunoreacted with antibodies against phospho-STAT3 tyr705 (left, rabbit monoclonal, 1:100; FITC-conjugated sheep anti- rabbit secondary, 1:200) and CD11b (right, as Figure 1 shows) at 12 h after EMF exposure (A-L). (**A-C**) Untreated cultures were used as a control. (**D-F**) Cultures pretreated with P6 (10 μM). (**G-I**) EMF induced more phosphorylation of STAT3 (green) and expression of CD11b (red) in N9 cells. (**J-L**) P6 inhibits the phosphorylation of STAT3 and expression of CD11b at 12 h after EMF exposure. (**M-O**) P6 pretreatment significantly suppresses the phosphorylation of STAT3, but a slight and significant increase of CD11b is still apparent at 3 h after EMF exposure. Scale bar 10 μm. (**P**) Bar graphs show semi-quantification of fluorescence intensity for p-STAT3 and CD11b in N9 cells. Results are presented as mean ± S.D. of three independent experiments. Statistical comparisons to control are indicated by * p < 0.05; ** p < 0.01.

It has been previously suggested that activated microglia express different proteins and surface markers. Of these, CD11b has the greatest biological significance [63,64]. Because increased expression of CD11b is a typical feature of microglial activation [65], we assessed the effect of EMF exposure on the expression of CD11b in N9 cells by FACS and confocal microscopy. EMF was found to significantly increase CD11b expression (Figure 1 &2I). Figure 1 clearly shows increases in CD11b expression by N9 cells 3 h and 12 h after EMF exposure (red line). A similar pattern was observed with immunolocalization and confocal microscopy. Immunofluorescence reaction was significantly increased 12 h after EMF exposure (Figure 2I). In contrast, no increase in CD11b expression was observed in the sham-exposure control groups (Figure 1, 2C &2F).

### Effect of EMF exposure on TNF-α, iNOS expression and NO release from N9 cells

**Figure 3:**
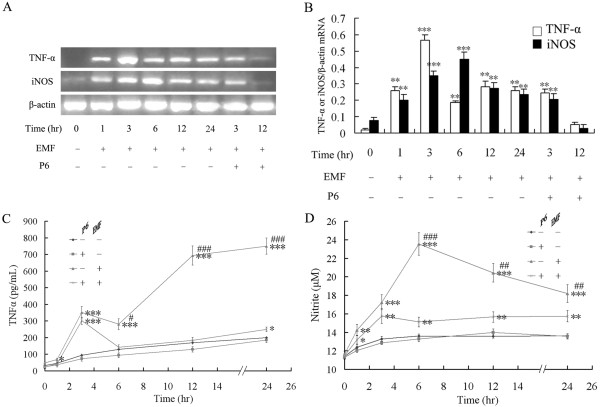
**EMF exposure induces TNF-α and iNOS expression and NO release from N9 cells**. Experiments were performed as described above. (**A**) RT-PCR analysis showing that EMF exposure significantly induces the mRNA expression of TNF-α and iNOS. (**B**) Mean ± S.D. of mRNA levels from five pooled samples per time point. (**C**) The concentration of TNF-α in the medium was determined by ELISA after cells were treated at the indicated time points. (**D**) The amount of nitrite was determined by the Griess reaction. Results are presented as the mean ± S.D. of five independent experiments. Statistical comparisons to control are indicated by * p < 0.05; ** p < 0.01; *** p < 0.001. Statistical comparisons between EMF treatment group and P6 preconditioning are indicated by # p < 0.05; ## p < 0.01; ### p < 0.001.

Given the pro-inflammatory effect of EMF exposure on microglia, we measured levels of TNF-α and iNOS, and the resulting NO production, in cell culture medium supernatants at the indicated times after EMF exposure. As shown in Figure 3A, EMF exposure significantly induced expression of TNF-α and iNOS. RT-PCR analysis showed that the levels of TNF-α and iNOS mRNA peaked at 3 h and 6 h, respectively, and were sustained up to 24 h after EMF exposure (Figure 3B). Because iNOS is an inducible enzyme, we examined its activity by measuring the amount of nitrite converted from NO in the medium using a Griess reagent. Release of NO was found to peak at 6 h and to remain high up to 24 h after EMF exposure (Figure 3D). Next, the secretion of TNF-α was measured by ELISA. Production of TNF-α reached its first peak at 3 h, gradually decreased, peaked again at 12 h and was then sustained for up to 24 h after EMF exposure (Figure 3C).

### Effect of EMF exposure on phosphorylation and DNA-binding activity of STAT3 in N9 cells

**Figure 4:**
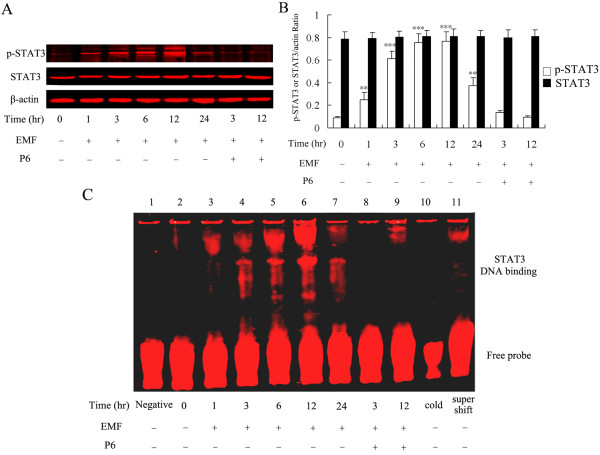
**EMF exposure induces expression of p-STAT3 and its DNA-binding activity**. Experiments were performed as described above. The protein samples were harvested at the indicated times after EMF exposure in N9 cells. β-Actin antibody was used as a control for equal protein loading. (**A**) Western blot analysis showing STAT3 activation with antibodies against phospho-STAT3 tyr705 and STAT3; p-STAT3 was labeled with green fluorescence and total STAT3 was labeled with red fluorescence. (**B**) The amount of p-STAT3 or STAT3/actin ratio was determined by densitometric analysis with software Image J 1.42. (**C**) STAT3 DNA-binding activity was measured by electrophoretic mobility-shift assays after EMF exposure. No nuclear extract was added to the labeled STAT3 probe (lane 1). EMF sham exposure was used as a control (lane 2). DNA binding was noted at 1, 3, 6, 12 and 24 h after EMF exposure (lane 3-7). P6 inhibited EMF-induced binding activity to STAT3 (lane 8-9). Unlabelled STAT3 was used as competitor DNA at a 200-fold molar excess (Lane 10). A super-shift assay was conducted by pre-incubating of nuclear extracts with anti-STAT3 (lane 11). Results are presented as mean ± S.D. of three independent experiments. Statistical comparisons to control are indicated by * p < 0.05; ** p < 0.01; *** p < 0.001.

In previous work, we have shown that activation of JAKs and STAT3 is involved in EMF-activated microglia [56]. To further determine the timing of STAT3 activation in EMF-stimulated microglia, we studied the immunolocalization, phosphorylation and DNA-binding activity of STAT3 at the indicated times. EMF exposure was found to result in strongly phosphorylated STAT3 in a time-dependent manner, with the peak activation occurring at 12 h (Figure 4A). The total amount of STAT3 did not change in response to EMF emission. Immunolocalization and confocal microscopy provided further evidence for STAT3 phosphorylation, showing a strong increase in fluorescence intensity in N9 cells at 12 h after EMF exposure (Figure 2G). In contrast, a low level of STAT3 phosphorylation was observed in the untreated (0 h) group (Figure 2A &4A).

Under basal conditions, STATs are located in the cytoplasm; however, when these transcription factors become phosphorylated, they translocate to the nucleus within minutes [66]. Accordingly, we performed gel mobility shift assays to analyze the ability of STAT3 to bind DNA. Figure 4C shows a specific DNA-protein complex that is slightly apparent at 1 h after EMF exposure, more fully apparent at 3 h and peaks at 12 h after EMF exposure. No protein-DNA complex formation was observed in the control group. The following experiments with the JAK inhibitor P6 were performed at the 3 and 12 h points.

### Effect of EMF exposure on JAK1 and JAK2 phosphorylation in N9 cells

**Figure 5:**
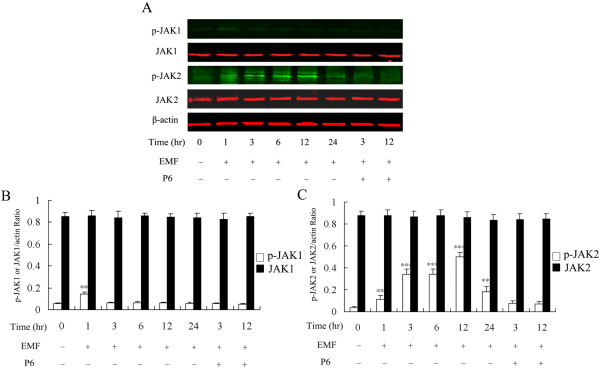
**EMF exposure induces phosphorylation of JAKs in N9 cells**. Experiments were performed as described above. (**A**) EMF exposure induces phosphorylation of JAK1 and JAK2 as determined by antibodies against phospho-JAK1 Tyr-1022/1023, JAK1, phospho-JAK2 Tyr-1007/1008 and JAK2. (**B**) The p-JAK1 or JAK1/actin ratio was determined by densitometric analysis. (**C**) The p-JAK2 or JAK2/actin ratio was determined by densitometric analysis. Results are presented as mean ± S.D. of three independent experiments. Statistical comparisons to control are indicated by * p < 0.05; ** p < 0.01; *** p < 0.001.

Given the above results, we focused our studies on the upstream tyrosine kinases of the STAT3 signaling molecule, *i.e.*, the JAKs [67], and performed a time course study of the phosphorylation of JAKs in EMF-stimulated N9 microglia. Western blots showed dramatic phosphorylation of JAK2 but not JAK1 in N9 cells at 1, 3, 6, 12 and 24 h after EMF exposure (Figure 5). Figure 5B shows a low level of JAK1 phosphorylation that occurred immediately at 1 h after EMF exposure; JAK1 phosphorylation subsequently returned to basal levels. In contrast, phosphorylation of JAK2 was found to increase over a period of several hours, showing kinetics similar to those of STAT3 phosphorylation (Figure 5C). JAK2 phosphorylation was also apparent at low levels in the control group. The total amount of JAK1 and JAK2 did not change in response to EMF stimulation.

### Inhibitory effect of P6 on EMF-induced CD11b, TNF-α and iNOS expression and NO release

To further assess the potential role of the JAK2-STAT3 pathway in EMF-induced activation and pro-inflammatory responses of N9 microglia, we examined whether the JAK inhibitor P6 could affect EMF-induced increase of TNF-α, iNOS and NO, and the initial activation of microglia. Western blot analysis and EMSA experiments show that P6 preconditioning completely blocks activation of JAK2 and STAT3 at 3 and 12 h after EMF exposure (Figure 4 &5). Our results also show that P6 preconditioning reduces CD11b expression (Figure 1B &2L), decreases expression of iNOS and TNF-α, and blocks NO release at 12 h after EMF exposure (Figure 3). Interestingly, the fluorescence intensity of CD11b was found to still be significantly increased at 3 h after EMF exposure even with P6 preconditioning (Figure 1A, 2O). In addition, P6 preconditioning was found to have a slight inhibitory effect on TNF-α and iNOS mRNA expression or protein synthesis at 3 h after EMF exposure (Figure 3).

## Discussion

In the present study, we observed N9 microglial activation and pro-inflammatory responses after EMF exposure. We found that the JAK2-STAT3 pathway is activated in EMF-stimulated N9 microglial cells. The activation of microglia and the secretion of pro-inflammatory factors were significantly reduced by P6 treatment at 12 h after EMF exposure, while P6 preconditioning did not inhibit the above processes at 3 h post exposure. Our data suggest that the JAK2-STAT3 pathway may play a pivotal role in the pro-inflammatory response but not in the initial activation of microglia after EMF exposure.

It has been reported that increased CD11b expression corresponds to extent of microglial activation [63,64,68]. Here, we observed a dramatic increase in CD11b expression in an *in vitro *model exposing N9 cells to 2.45 GHz using a waveguide system that simulates occupational or residential exposure, at a specific absorption rate (SAR) of 6 W/kg. This result suggests that EMF may potentially affect microglial activation. Our data are consistent with earlier findings demonstrating increased glial reactivity in a model using about 900 MHz from a global system for mobile communication, at a high SAR of 6 W/kg [17,18,69]. Other investigators, however, have argued that EMF exposure does not lead to microglial activation at low SAR values simulating either microwave radiation or mobile telephone radiofrequency fields [70-72]. Taken together, these studies show microglial reactivity only in model systems with high SAR values of up to 6 W/kg. Thus, we consider that high-energy EMF, with definite thermal effects [73], could potentially induce microglial activation.

Microglia are known to be exquisite sensors of even minor pathological changes in the CNS [28,61]. They also act as active contributors to neuronal damage in neurodegenerative diseases such as Alzheimer's disease, Parkinson's disease and HIV dementia [74,75]. An increasing amount of evidence suggests that migroglia are key factors in the process of neuroinflammation [74]. Microglia-induced neuronal injury may be mediated by the production of TNF-α, NO, and reactive oxygen species [76-78]. Our results confirm significant up-regulation of TNF-α and iNOS mRNA, and release of the pro-inflammatory factors TNF-α and NO, in N9 microglia after EMF exposure. These results suggest that EMF, as an external physical factor, could facilitate microglia pro-inflammatory responses through the secretion of pro-inflammatory factors. This activity may ultimately contribute to CNS impairment or disease.

It is well known that microglia monitor the external environment [61] and respond to external stimuli [79] via signaling cascades that allow them to perturb membrane function and trigger the activation of one or more intracellular signaling pathways [75]. In contrast, there is a lack of information regarding signal transduction mechanisms and molecular targets of EMF-activated microglia. Here, our time course experiments show different expression levels of the JAK-STAT pathway in EMF-activated microglia. It has been demonstrated that the JAK-STAT cascade plays an essential role in driving a variety of immune responses in glial cells in the brain [54,80]. Different expression levels of the JAK-STAT pathway have been detected in glial cells in the brain [51-53,76,80-82] and associated with pathological CNS conditions such as cerebral ischemia[52,55,83], traumatic brain injury [84,85] and brain inflammation [86]. These observations suggest that EMF exposure likely affects microglial activation through the activation of the JAK-STAT pathway.

To investigate the potential function of the JAK-STAT pathway in EMF-activated microglia, we next examined whether the JAK inhibitor P6 could affect the EMF-induced increases of TNF-α, iNOS, NO and CD11b. P6 can effectively block the activation of JAK1, JAK2 and STAT3 [87,88]. Our results revealed that the activation of JAK2 increased with kinetics similar to those of phosphorylated-STAT3. The activation of JAK2 and STAT3 was significantly inhibited by P6 at 3 and 12 h after EMF exposure. These results provide further evidence that JAK2-STAT3 signaling plays a role in the reactivity of EMF-stimulated microglia.

Most previous studies have shown that the JAK-STAT signaling pathway is involved in microglial activation [50-55]. In our study, the activation of microglia, the transcription of TNF-α and iNOS, and the secretion of TNF-α and NO were not significantly inhibited at 3 h by P6 in EMF-activated microglia, however. These results suggest that the JAK2-STAT3 pathway may not mediate the initial activation of microglia after EMF exposure. Other signaling pathways may be involved in this process. Chang *et al*.[89] reported that microglial inactivation by ketamine is at least partially due to the inhibition of ERK1/2 phosphorylation. Ryu *et al*. [90] reported that thrombin induces NO release from cultured rat microglia via protein kinase C, mitogen-activated protein kinase, and NF-kappa B. Thus, we speculate that EMF exposure activates microglia through other signaling pathways.

It has been demonstrated that activated microglia secrete a diverse range of pro-inflammatory and neurotoxic factors such as superoxide, TNF-α, interleukin (IL)-1β, IL-6 and NO [74]. The cytokines IL-1β and TNF-α, as mentioned above, may stimulate microglia to produce monocyte chemoattractant protein (MCP)-1, macrophage inﬂammatory protein (MIP)-1α, and MIP-1β, which also may contribute to neuroinflammation [91]. After becoming activated by cytokines, microglia also release more cytokines into the extracellular space, thus forming an autocrine loop with positive feedback between microglial activation and cytokine production. This loop could explain the maintenance of microglial activation and the enhancement of pro-inflammatory responses for 24 h after EMF exposure. Several reports have reported that STAT3 acts as a transcription factor in modulating cytokine-induced pro- and anti-inflammatory responses [92,93]. Recently, Tanabe *et al. *[94] reported that TNF-α induces IL-6 synthesis through the JAK/STAT3 pathway in rat C6 glioma cells. Mir *et al. *[95] indicated that the enhancing effect of TNF-α on IFN-γ induced iNOS/NO generation is dependent on the JAK-STAT signaling pathway. In this study, P6 was found to reduce CD11b expression, decrease the expression of TNF-α and iNOS, and relieve the release of TNF-α and NO at 12 h in EMF-activated microglia. Our data suggest that a feedback loop may be formed to maintain the activation of microglia and extend the pro-inflammatory responses through the JAK2-STAT3 pathway. Based on these data, we hypothesize that after EMF exposure: (i) there might be some other signaling pathway that rapidly activates microglia; (ii) pro-inflammatory factors secreted by activated microglia may activate the JAK-STAT pathway; and (iii) the activated JAK-STAT signaling pathway may further induce release of pro-inflammatory factors and maintain the activation of microglia.

We studied the effects of EMF exposure on cultured N9 microglial cells and demonstrate that an initial activation of microglia is induced by EMF exposure. In addition, many other physical factors such as infrasound exposure, irradiation, heat shock treatment and hyperthermia, can stimulate activation and pro-inflammatory reactions of microglia [96-100]. The transmembrane signal transduction mechanisms of microglial activation in these physical environments remain poorly understood. Further investigations into these transmembrane signal transduction mechanisms may help to protect humans against electromagnetic radiation, ionizing radiation and other hazardous physical factors.

## Conclusions

Our results strongly suggest that external electromagnetic emission as a physical stimulation directly triggers an initial activation of microglia and produces significant pro-inflammatory responses. Activation of JAK2-STAT3 signaling occurs in parallel with the microglial activation and the release of pro-inflammatory factors. Microglial activation and pro-inflammatory responses are significantly reduced by P6 at 12 h, but not at 3 h, after EMF exposure. These results suggest that the JAK2-STAT3 pathway may not mediate the initial microglial activation, but does promote pro-inflammatory responses in EMF-stimulated microglial cells. Our results provide a basis to determine whether the pro-inflammatory responses of EMF-stimulated microglia can be suppressed by inhibition of the JAK2-STAT3 pathway in therapeutic interventions.

## List of abbreviations

EMF: Electromagnetic fields; NO: nitric oxide; TNF-α: tumor necrosis factor-α; JAK-STAT: Janus kinase-signal transducers and activators of transcription; iNOS: inducible NO synthase; P6: Pyridone 6; FACS: fluorescence activated cell sorting; EMSA: electrophoresis mobility shift assay; ELISA: enzyme-linked immunosorbent assay.

## Competing interests

The authors declare that they have no competing interests.

## Authors' contributions

This study is based on an original idea of XSY. XSY and GLH wrote the manuscript. XSY and MQL carried out the FACS and confocal double-label immunofluorescence assays. GLH, YTH, CHC carried out the RT-PCR, western blotting and EMSA assays. YW carried out some mediator assays. GBZ provided EMF exposure system. All authors have read and approved the final manuscript.
